# Ciprofloxacin-Resistant *Salmonella enterica* Serotype Kentucky Sequence Type 198

**DOI:** 10.3201/eid2005.131575

**Published:** 2014-05

**Authors:** Regan Rickert-Hartman, Jason P. Folster

**Affiliations:** Centers for Disease Control and Prevention, Atlanta, Georgia, USA

**Keywords:** antimicrobial resistance, Salmonella, *Salmonella enterica* serotype Kentucky, salmonellae, ciprofloxacin, serotype, bacteria

**To the Editor:** Mulvey et al. (*1*) reported the emergence of ciprofloxacin resistance in *Salmonella enterica* serovar Kentucky of multilocus sequence type 198 (ST198) in Canada (*1*). Ciprofloxacin resistance in *S. enterica* ser. Kentucky was reported in 2011 in patients from Europe, most of whom had traveled to Africa and the Middle East (*2*). Since then, *S. enterica* ser. Kentucky ST198 with additional resistance to third-generation cephalosporins and carbapenems has been reported from France and Morocco, again associated with travel (*3*). Poultry has been implicated as the most likely vehicle for infection by this sequence type (*2*,*3*). Resistance to third-generation cephalosporins and carbapenems has not been seen in North America; however, the emergence of ciprofloxacin-resistant infections has been observed (*1*). 

In the United States, *S. enterica* ser. Kentucky is the most common serotype isolated from chickens and the second most common found among retail chicken, but ciprofloxacin resistance has not been documented among these sources (*4*). We sought to determine if ciprofloxacin- or ceftriaxone-resistant *S. enterica* ser. Kentucky has emerged in humans in the United States. We examined isolates and data from the National Antimicrobial Resistance Monitoring System to document antimicrobial resistance and sequence type and to assess possible risk factors for acquiring infection.

Participating state and local public health laboratories submit every 20th nontyphoidal *Salmonella* (NTS) isolate to the Centers for Disease Control and Prevention for susceptibility testing. MICs of >15 antimicrobial agents were determined by using broth microdilution (Sensititer, Cleveland, OH, USA) according to the manufacturer’s instructions. Where available, Clinical and Laboratory Standards Institute performance standards were used for interpretation of MICs; otherwise, interpretations established by the National Antimicrobial Resistance Monitoring System were used (*5*,*6*).

During 2009–2012, a total of 21 (0.2%) of the 9,225 NTS isolates tested were *S. enterica* ser. Kentucky. Six (29%) were resistant to ciprofloxacin; all were susceptible to ceftriaxone (Table) (*5*). As was observed in Canada, the 6 resistant isolates were >80% similar by pulsed-field gel electrophoresis analysis (*Xba*I; data not shown), and all 6 were ST198. Although a rare cause of human infection, *S. enterica* ser. Kentucky represented 23% (6/26) of all ciprofloxacin-resistant NTS detected during 2009–2012. 

**Table:** Patient and isolate information for 6 cases of infection with ciprofloxacin-resistant *Salmonella enterica* serotype Kentucky sequence type 198 detected by the National Antimicrobial Resistance Monitoring System, United States, 2009–2012*

Isolate ID	Patient age, y/sex	Patient race	Patient travel history	Year specimen collected	Specimen type	Antimicrobial resistance†
AM41047	<1/F	Black	Ethiopia	2009	Stool	AMP, FIS, GEN, NAL, STR, TET
AM45820	54/F	Unknown	Unknown	2010	Urine	AMP, COT, FIS, GEN, NAL, STR, TET
AM47052	56/F	Asian	India	2011	Urine	AMP, FIS, GEN, NAL, STR, TET
2012AM-1081	2/F	Asian	India	2012	Stool	AMP, NAL
AM50773	37/M	Asian	India	2012	Stool	AMP, AUG, FIS, GEN, NAL, STR, TET
2012AM-0353	42/F	White	Saudi Arabia	2012	Urine	AMP, FIS, FOX, KAN, NAL, STR, TET

*ID, identification; AMP, ampicillin; AUG, amoxicillin-clavulanic acid; COT, trimethoprim-sulfamethoxazole; FIS, sulfisoxazole; FOX, cefoxitin; GEN, gentamicin; KAN, kanamycin; NAL, nalidixic acid; STR, streptomycin; TET, tetracycline. †Resistance of isolate from infected patient to antimicrobial agents other than ciprofloxacin.

The median age of the 6 patients with ciprofloxacin-resistant *S. enterica* ser. Kentucky infections was 32 years (range 9 months–56 years); 5 (83%) were female. Of the 4 patients for whom information was available, 2 were hospitalized, and 1 died. Specimen sources were stool (n = 3) and urine (n = 3). Travel histories were obtained for 5 patients, and all had traveled internationally in the 7 days before specimen submission: 2 were residents of other countries (Saudi Arabia and Ethiopia), and 3 were US residents who had returned from travel to India. By comparison, only 3 of 10 patients with ciprofloxacin-susceptible infections had traveled (p = 0.02).

Resistance to ciprofloxacin in *Salmonella* is a growing concern because it limits treatment options for invasive disease. We describe ciprofloxacin-resistant *S. enterica* ser. Kentucky isolated from 6 patients in the United States. The emerging global story of *S. enterica* ser. Kentucky ST198 demonstrates the need for international integration of surveillance for antimicrobial drug resistance.
